# Oral Health and Dental Health Care Experiences of Patients From the Netherlands With Parkinson's Disease: A Qualitative Study

**DOI:** 10.1111/ger.12822

**Published:** 2025-05-03

**Authors:** Merel C. Verhoeff, Karina E. Pigeaud, Danisha M. Tholen, Miranda Rezk, Frank Lobbezoo

**Affiliations:** ^1^ Department of Orofacial Pain and Dysfunction Academic Centre for Dentistry Amsterdam (ACTA), University of Amsterdam and Vrije Universiteit Amsterdam Amsterdam the Netherlands

**Keywords:** education, oral health, Parkinson's disease, patients voice, practice management, qualitative research

## Abstract

**Background:**

Parkinson's disease patients have poorer oral health than their healthy peers. Insight into their own experiences is vital for improving dental care.

**Objective:**

To better understand the experiences of Parkinson's disease patients' with their oral health and dental care.

**Materials and Methods:**

Eleven semistructured interviews with Parkinson's disease patients from the Netherlands were conducted. Data were analysed using thematic analysis to identify recurring themes and patterns.

**Results:**

Participants' narratives revealed challenges in accessing dental treatment, primarily due to motor and communication difficulties. They also highlighted organisational barriers, such as appointment scheduling and awareness of care practitioners about Parkinson's disease patients' oral health. Furthermore, they expressed the urgent need for Parkinson's disease‐specific dental education to (dental) care practitioners and research initiatives that focus on Parkinson's disease patients' oral health and dental care.

**Conclusion:**

Parkinson's disease patients' experiences highlight the need for tailored care interventions and improvements in dental care systems.

## 1 Introduction

Parkinson's disease is a neurodegenerative disease with a rapidly growing global prevalence of 6.1 million people [1, 2]. It is a complex condition encompassing motor (e.g., tremors, bradykinesia) and nonmotor (e.g., loss of smell, cognitive decline, pain) symptoms, each with highly individualised patterns [3, 4, 5]. While there is currently no cure for Parkinson's disease, symptom management is possible with pharmacotherapy and treatments such as physiotherapy, surgery, or speech therapy [6].

Although the oral health of patients with Parkinson's disease patients is understudied, it is receiving increased attention from researchers. Previous studies have revealed that Parkinson's disease patients typically experience poorer oral health [7], increased pain, and more dysfunction of the orofacial area than healthy individuals [8]. Furthermore, subjectively perceived and objectively measured salivary problems are more frequently found among people with Parkinson's disease than in healthy individuals [9].

The current literature is expanding on oral health and dental care in Parkinson's disease patients. For example, clinicians describe their experiences of treating these patients [10], as complex and suggest that reducing the bureaucratic burden and improving our knowledge on oral health in Parkinson's disease patients could assist the dental care practioner [10]. The voices of the patients themselves have yet to be explored. This is surprising because it is impossible to improve oral health and dental care without including the experiences of the target group, and authors have suggested that patients' experiences with oral health and dental care need to be more comprehensively studied [11, 12]. Accordingly, this study aimed to better understand the oral health and dental care experiences of people with Parkinson's disease the findings of which could substantially enhance patient care.

## 2 Material and Methods

This research took a qualitative approach using semistructured interviews. This enabled the exploration of predetermined topics while also allowing for new topics and insights to be addressed [13, 14]. Furthermore, this design offered methodological flexibility and facilitated a more profound comprehension of individual perspectives than a quantitative approach would have achieved [13, 14].

People living in the Netherlands with Parkinson's disease, who were able to speak with the interviewers, were eligible to participate. Those without Parkinson's disease, with Parkinsonism, or individuals with personal or professional ties to the interviewers were excluded. Participants were recruited through an advertisement on LinkedIn (LinkedIn Corporation, Sunnyvale, California, U.S.), and promoted through social media channels and the newsletter of the very active, nationwide Dutch Parkinson's Disease Association (Parkinson Vereniging; https://www.parkinson‐vereniging.nl/). Participants provided written informed consent prior to data collection. The study was approved by the Ethical Committee of ACTA (approval number 2023–31015).

The study's methodology was based on Ritchie & Lewis (2003) [14], which was used by Verhoeff et al.'s [10] to investigate dental care practitioners' perspectives. For the current study, this methodology was adapted to suit people with Parkinson's disease patients [10]. Guided by a semistructured interview schedule (Table S1) participants were asked questions that focused on four main themes: (1) ‘oral hygiene’; (2) ‘dental treatments’; (3) ‘organisation’; and (4) ‘education’ and research. The interviews were conducted by two third‐year female dental master students (DT, MR) at a location, date and time convenient for the participants, either in person or via Zoom (Zoom Video Communications Inc., San Jose, California, U.S.). Interviews ranged from 30 to 60 min, depending on the comprehensiveness of the participants' input. The interviews were recorded and transcribed verbatim, and any information that could identify the participants was removed. Participant inclusion continued until no new topics or insights arose from the interviews [14]. Two additional participants were included to ensure comprehensive coverage, with an anticipated total of 10 to 15 participants. Following the interviews, the transcripts were analysed thematically [10, 13] by three researchers using the ATLAS software program.ti (Scientific Software Development GmbH, Berlin, Germany). A discussion took place with a fourth researcher during and after analysis to confirm the results.

## 3 Results

All interviews were conducted between 1 May and 1 July 2023, via Zoom. No new topics or insights emerged after interviewing nine participants. Subsequently, data saturation was confirmed by adding two additional interviewees. In total, 11 participants were interviewed, of which seven were male. On average, participants were 68 years old and had their diagnosis of Parkinson's disease confirmed for just over 7 years prior to being interviewed. Results are presented by the main themes in **bold**, and subthemes presented throughout an underscore. Participant quotes are translated from Dutch to English, and checked by a bilingual researcher. Table 1 presents the results.

**TABLE 1:** Main themes, subthemes and summary of the Parkinson's disease patient's vision in respect of these (sub)themes.

Main themes	Subthemes	Summary
1. Treatment	Oral Health	Some Parkinson's disease patients experience difficulties with oral hygiene because of Parkinson's disease‐related factors (e.g., reduced motor skills, rigidity of facial muscles, lack of energy and motivation). Most Parkinson's disease patients find electric toothbrushes helpful and easier to use. Parkinson's disease patients experience salivary problems, before the diagnosis (viz., dry mouth), and after the diagnosis (e.g., dry mouth, salivary loss). But also swallowing difficulties.
	Dental visits	Parkinson's disease patients have mixed experiences on changes in the frequency and type of dental visits and treatments before and/or after diagnosis. Parkinson's disease patients experience dental visits as too long and energy‐demanding. Parkinson's disease patients experience stress before and during dental consultation, and exacerbating symptoms as a consequence. Parkinson's disease patients explain that proactive behaviour from themselves is needed in care settings. When dental care practitioners instruct Parkinson's disease patients, it is often too much information and rushed. Therefore, the information is not well received or insufficient. Parkinson's disease patients experience little interest from dentists on the disease itself. Flexible cancellation of dental visits, when having Parkinson's disease, is needed. Dental chairs are often experienced as uncomfortable.
2. Organisation	Collaboration	Parkinson's disease patients experience a good intercollegial consultation within dental practices. Parkinson's disease patients do not experience collaboration between dental and other care practitioners. More exchange of information and better intercollegial consultation would be appreciated, although some opposed this, fearing it would increase the workload of their care practitioner.
	Accessibility	Travel time is a barrier to seeking dental consultation and treatment for Parkinson's disease patients. Parkinson's disease patients are unaware that dentists experienced in geriatrics may be included in ParkinsonNet, and thus is their accessibility lacking. Some Parkinson's disease patients need financial support for increasing dentals costs due to worsening oral health. Often there is not enough room in dental practices to bring aids (e.g., walkers).
	Points of improvement care system	Half of the participants state that dental care for Parkinson's disease patients should be included in the primary care system. Parkinson's disease patients would like more time and rest during dental care; dentists are perceived as being rushed. Good communication and consultation with Parkinson's disease patients are needed, so that they can prepare themselves for, e.g., treatments. More attention should be paid on prevention (e.g., frequency of dental visits, oral hygiene instructions and additional fluoride). Attention for the mouth should be considered in guidelines on multidisciplinary collaboration in Parkinson's disease.
3. Education and research	Knowledge	Parkinson's disease patients experience a lack of knowledge about Parkinson's disease in just graduated young dentists. Parkinson's disease patients are not aware of possibilities for referral to a dentist specialised in geriatrics.
	Points of improvement for education	Dental education should emphasise what Parkinson's disease entails, the different manifestations of the disease, and the effects of disease presentation on dental treatments.
	Research suggestion	How to cope with Parkinson's disease‐related jaw tremors (during dental visits)? Is dry mouth present as a nonmotor symptom before and/or during the Parkinson's disease diagnosis? Are people experiencing unexplained orofacial pain before the Parkinson's disease diagnosis? Is periodontitis associated with Parkinson's disease? Are management decisions made by a dentist different in case of Parkinson's disease patients from those for healthy peers? And why? Parkinson's disease medication is dissolvable; residues are present in the mouth. Is that bad for the oral environment? Are salivary stones more present due to dry mouth problems in Parkinson's disease?

## 4 Treatment

### 4.1 Oral Health

Some participants did not experience motor skill problems associated with their oral hygiene routine (e.g., teeth brushing, interdental care) after being diagnosed with Parkinson's disease, and their routine stayed the same. However, some participants noticed that they need to pay more attention to it: ‘*What I do more now is consciously brushing my teeth and paying close attention to it, because I know that I am more at risk’*. This group had difficulties with oral hygiene due to deteriorating motor skills, tremors and rigidity of the facial muscles. One participant also mentioned: ‘*It is not always only motor skills that deteriorate; it is often my energy level that is not suitable enough*’. Most participants said they used an electric toothbrush to overcome motor difficulties as this requires less movement. Other aids, such as mouthwash and toothpaste with more fluoride, were also mentioned to maintain good oral health. However, participants also mentioned that the deteriorated motor skills are, in the early phase, not always a big problem: ‘*In my case, I have a tremor at the left side, but my hand preference is right. Therefore, I don't experience many problems during my dental care routine, yet’*.

Participants reported having experienced problems with saliva, both too much saliva and having a dry mouth, causing, among other issues, problems with swallowing and difficulties with communication: ‘*I have a lot more saliva in my mouth. If I bend down, it runs out of my mouth. And when I am around others, I have to swallow more often; it is not comfortable*’. Furthermore, participants said they had experienced a decline in their oral health, with problems such as caries, periodontal decline and unexplained pain, since the diagnosis or even a short period prior to diagnosis: ‘*I had a good oral health, and suddenly I had four cavities*. *No one understood’*. They attributed the decline in their oral health with salivary changes, polypharmacy that can cause salivary changes, and their deteriorating motor skills (Table 1).

### 4.2 Dental Visits

Most participants reported that the frequency of dental visits or treatments had not changed following their diagnosis, although three participants mentioned having increased dental visits (to the oral hygienist and/or dentist) due to declining oral health. Participants typically emphasised that dental visits are exhausting and stressful, and to avoid additional stress, they preferred not scheduling other appointments on the same day as other appointments: ‘*I can get very stressed from one appointment. I avoid several appointments on the same day, so I will not schedule something else when I go to the dentist*’. They also noted that dental visits, particularly with dentists, are rushed and that they would benefit from having more time for their treatment and to rest from their appointment. Also, a busy atmosphere in the waiting room can also trigger stress and thus exacerbate Parkinson's disease symptoms (Table 1).

Participants commented that there is often insufficient space in the treatment room to manoeuvre and accommodate aids such as a walker. One patient expressed, ‘*When I come in with my walker, I bump into everything and feel very unhappy. Then, you really feel like a disabled person’*. Moreover, participants thought that treatment times are too long for patients with Parkinson's disease. They said they found it a challenge to keep their mouth open and lie in a dental chair for an extended period of time. The interviewees emphasised the importance of dental staff managing appointment times efficiently and discussing treatment times with patients with Parkinson's disease, so that they can prepare themselves. Moreover, interviewees mentioned that it would help them if employees of dental offices understood and accommodated the need to cancel appointments when not feeling well, without facing social and financial consequences.

Finally, participants experienced little interest from dentists of the disease process and its influence on dental treatments. Participants explained that they had to be proactive with dental care practitioners: ‘*I even have the idea that my dentist sometimes forgets that I have Parkinson's disease*’, although another participant pointed out the positive side of it: ‘*Sometimes, it is also nice to just be a normal person instead of a Parkinson's disease patient’* (Table 1).

## 5 Dental Care Organisation

### 5.1 Collaboration

Study participants experienced good collaboration within dental practices, among the dentists and dental hygienist, however, they were not asked about their oral health by other health care practitioners involved in their management of their Parkinson's disease (e.g., nurses, neurologists). In addition, participants said that there appeared to be no collaboration or discussions about oral health issues between their dental care practitioners and other health care practitioners managing their Parkinson's disease. One participant said that everyone seemed to work separately without interacting with each other, *‘Everyone works in his/her own silo with tunnel vision’*.

Some participants also noticed that sometimes medical treatments are initiated, but their dental care practitioner is not notified: ‘In the hospital, they initiated Botox therapy for my salivary problems. By coincidence, I mentioned it to my dentists. He had no idea, but he explained the association between the salivary flow and my oral health. I was stunned’.

The majority of the interviewees expressed a desire for more information exchange and improved collaboration among all health care professionals, suggesting the establishment of multidisciplinary centres where all health care practitioners involved in Parkinson's disease management can communicate and access each other's data. However, one participant opposed the idea, stating, ‘I do not want my neurologists to ask about my oral health or that they have to collaborate together. That is all taking up valuable time’. This may indicate that patients could experience the work pressure of her (dental) care practitioners, which, in this example, she wanted to protect (Table 1).

### 5.2 Accessibility

Most of the participants mentioned that travel time was a barrier for seeking dental care, especially when seeking specialised treatments from geriatric dental specialists as they had to travel longer distances to see them. In addition, accessibility was not optimal because most participants were unaware of the existence of dental specialists until they were recently included in ParkinsonNet (the specialised care network for Parkinson's disease and Parkinsonism patients in the Netherlands) (Table 1).

### 5.3 Points of Improvement

All participants were living at home and did not experience any difference in their health insurance coverage for dental care following diagnosis. In the Netherlands, this means that they have to pay for dental care themselves, unlike institutionalised patients who receive subsidised care. About half of the participants did not find this to be a problem and were used to paying for dental care. Some participants expressed a desire for financial compensation specifically for people with Parkinson's disease who live at home. They suggested that dental care should be included in the primary care system for the Dutch population, including those with Parkinson's disease. These interviewees believed that dental care is important for people with Parkinson's disease, given that their deteriorating motor skills increases the need for affordable dental care. They said, ‘*We need extra care because activities like brushing become more difficult, and treating us takes more time’* (Table 1).

## 6 Education and Research

### 6.1 Knowledge

Study participants observed that general dentists have limited knowledge about Parkinson's disease, and how to address treatment difficulties related to Parkinson's disease symptoms such as tremors and rigidity.

During the interviews it became clear that the participants themselves also had limited knowledge about the known associations between Parkinson's disease and oral health, and about options for dental care associated with Parkinson's disease. Some interviewees were not aware that there are dentists specialising in geriatrics, including Parkinson's disease care. When informed of these specialised dentists, they expressed interest in seeking dental care if their Parkinson's disease symptoms worsened and their current dentist could not provide adequate treatment: ‘I first want to know what that specialised dentist can do for me, as compared to my own dentists’. They emphasised that their good and trusted relationship with their current dentist is of utmost important to them and would discourage them from seeking dental care elsewhere (Table 1).

### 6.2 Points of Improvements

The interview participants proposed several ways to enhance the education of dental students in treating patients with Parkinson's disease, including understanding the symptoms of Parkinson's disease, the impact of these symptoms on dental treatments (e.g., rigidity, tremors and stress sensitivity), and how dental care practitioners can adjust their approach. In addition, they suggested that internships with Parkinson's disease nurses and/or neurologists, and patient demonstrations, could upskill dental students' interactions with Parkinson's disease patients and/or neurologists: ‘It could be helpful to invite lecturers to discuss Parkinson's disease, including its impact on dental care, to make it less abstract’ (Table 1).

### 6.3 Research Suggestions

Participants were asked about what further areas of research should be undertaken. They raised several questions, including ‘*I developed periodontitis before the Parkinson's disease diagnosis; is that associated?*’, ‘*I dissolve some of my medication, so residues are present in my mouth after drinking the liquid, which is possibly not good for my teeth. Should it be better to sort that out and give advice on that matter?*’, and ‘*I have a dry mouth, and after that, I also developed saliva stones, which is really painful. Has that something to do with Parkinson's disease*?’. Others sought more practical questions, such as researching excessive movements when lying in the dental chair and how to overcome them to get better dental help, and developing aids to overcome movement difficulties with oral self‐care. Finally, one of the participants asked if research had been conducted to investigate if the management decisions made by a dentist are different in the case of Parkinson's disease patients from those for healthy individuals. The participant's motivation for this curiosity was: ‘I often feel that my dentist treats me differently because of my Parkinson's disease, which is, of course, normal because I have special needs in comparison to healthy persons. However, I wonder if the dentist also thinks: is he still worth it?’ (Table 1).

## 7 Discussion

This study aimed to better understand Parkinson's disease patients' experiences with their oral health and dental care. The interviewees raised several challenges in seeking and receiving dental care and suggested improvements in their treatment, dental care organisation, education and research.

### 7.1 Treatment

Participants highlighted the challenges Parkinson's disease patients face in maintaining oral health and visiting dental care practitioners. Some participants reported minimal changes in their oral hygiene routine, while some experienced difficulties due to deteriorating motor skills, increased salivary issues, decreased energy levels and the need for additional aids, such as electric toothbrushes and fluoride toothpastes. Parkinson's disease patients recognise that the peripheral side affected by Parkinson's disease is important in relation to hand preference; especially at the early phase of Parkinson's disease, where unilateral and axial involvement is present as opposed to the later stages of the disease where bilateral involvement is present [15]. However, to the best of our knowledge, hand preference has not been investigated.

Participants mentioned that dental visits caused stress for Parkinson's disease patients due to physical and emotional strain, and having logistical issues when accessing dental care, such as inadequate space for mobility aids and insufficient time for patient care. These findings align with those of previous research with dental care practitioners [10]. Notably, this could increase difficulties with dental health care since stress sensitivity is higher in Parkinson's disease patients, and stress also increases Parkinson's disease‐related symptoms. However, the difficulty is that some patients also experience tremor during rest, which can be aggravated by lying in the dental chair as relaxed as possible.

### 7.2 Dental Care Organisation

Participants discussed collaboration inside and outside the dental care practice, the accessibility of the dental care practice (physically, financially), and provided suggestions for improving dental care organisation.

Our participants were satisfied with the collaboration among staff in dental offices; however, interdisciplinary collaboration was found to be lacking. Communication issues between dental and other care practitioners were also a concern, highlighting the need for more coordinated care and information exchange, so that the dental team is informed correctly about the disease progression and difficulties of the patient, including medication usage. And vice versa, when oral health is at risk, general health may also be worsened. This is also mentioned in earlier statements; however, this is still an enormous challenge [16].

In the Netherlands, access to specialised dental care remains challenging for people with Parkinson's disease, primarily owing to a lack of awareness about available services. While some participants were content with paying for dental care out of pocket, others suggested adjustments to insurance coverage to include Parkinson's disease‐specific dental care needs. This aligns somewhat with previous research that found that dental costs are higher for Parkinson's disease patients living at home and that the related health policy is insufficient [10]. Furthermore, our participants reported that their good and trusted relationship with their current dentist is paramount. It is possible that when their dentist is no longer capable of helping this patient group, Parkinson's disease patients get lost and are not receiving proper dental care anymore. It may be helpful to work with a bridging period, during which Parkinson's disease patients can work on building a relationship with a specialised dentist, but at the same time are seen by their regular dentist. The large shortage of dentists in the Netherlands is a disadvantage in guaranteeing such a period.

Interviewees mentioned that they have to be proactive and take control of their own health care needs. In people with Parkinson's disease, motivation and energy levels are not optimal [17, 18]; this disrupts the circuits affecting motivation. Is it then fair to design a health care system where proactiveness is needed to receive sufficient health care? Watt (2007) [19] stated that not only downstream interventions (i.e., the responsibility of the individual, clinical prevention and health education of the individual) are needed to receive sufficient health care, but also upstream interventions (i.e., health policy) are needed [19]. In this target group, more and improved upstream interventions are needed, in consultation with Parkinson's disease patients themselves, to overcome the difficulties of the disease.

### 7.3 Education and Research

Participants stressed the need for enhanced training for dental professionals on Parkinson's disease‐related challenges and a desire for more research into the specific oral health issues Parkinson's disease patients face. They proposed integrating Parkinson's disease education into dental curricula and exploring practical solutions for Parkinson's disease patients' unique dental care needs. Although this is a good suggestion for improving dental care in Parkinson's disease patients, it is possibly not feasible to learn about all different complex disorders since many patients present themselves in general practices. However, basic knowledge should be increased. Furthermore, in the Netherlands, there have been discussions on shortening the country's dental curricula. Nevertheless, feasible solutions to make health care in people with complex diseases valuable should be considered to enhance good quality health care.

It became clear that many interviewees are not aware of the specialised dentists, who are more knowledgeable of the needs of Parkinson's disease patients. Furthermore, participants were not aware of the existing scientific evidence on oral health in relation to Parkinson's disease. This demonstrates that the findings of research on oral health themes to date have not been successfully disseminated to people with Parkinson's disease. This suggests that researchers and/or dental care practitioners do not effectively communicate this information to patients. More attention should be paid to the valorisation of all stakeholders involved.

## 8 Limitations of the Study and Recommendations for Future Studies

In the current study, only Parkinson's disease patients themselves were included. Although the participants had a reasonable degree of disease insight, care partners may see some situations differently. Furthermore, the participants were living at home. It is expected that institutionalised Parkinson's disease patients in the Netherlands have a different view on oral health and dental care, since this is taken care of differently. *This study only concerned Parkinson's disease patients living in the Netherlands, therefore, the findings are unlikely to be generalisable to people with Parkinson's disease in other countries. Nevertheless, it is possible that the findings will be of interest to, and inform others with Parkinson's disease and practitioners working in this area of health care*. In addition, the participants in this study were not yet in the late stages of the disease process, which reflects a limited patient experience. The discussion during the interview and conclusions drawn are interpreted by the authors, based on what the interviewees reported. The transcripts were not returned to participants for member checking, but only when it was not clear what participants meant. Consequently, there may be some interpretation bias. This article forms the basis for further elaboration in future studies. We recommend including focus groups, a strategy that holds promise for comprehensive and insightful discussions [20]. By involving Parkinson's disease patients, care partners and even their dental care practitioners, we can gain a wealth of knowledge on several topics related to oral health and dental care.

## 9 Conclusion

This qualitative study showed the challenges faced by Parkinson's disease patients in maintaining oral health and accessing dental care. Overall, the study emphasises the importance of addressing Parkinson's disease patients' multifaceted dental care needs through improved guidance, clinical practices, enhanced education and targeted research.

## Author Contributions

M.C.V. and F.L. developed and designed this study. D.M.T. and M.R. performed and transcribed the interviews. M.C.V., D.M.T., and M.R. analysed the interviews and discussed them with F.L. when applicable. M.C.V. drafted this manuscript. Finally, all authors reviewed the manuscript and approved the final version.

## Conflicts of Interest

F.L. reports grants and others from Sunstar Suisse SA, grants from SomnoMed, grants from Vivisol‐ResMed, grants from Health Holland/TKI, and grants from NWO, outside the submitted work.

## Supporting information


**Table S1.**The main domains of the topic guide. These domains were only used to semistructure the interviews.

## Data Availability

The data that support the findings of this study are available from the corresponding author upon reasonable request.
