# Supplementary material for: Oral Health and Dental Health Care Experiences of Patients From the Netherlands With Parkinson's Disease: A Qualitative Study
Source: Gerodontology. 2025 May 3;42(4):529–35. doi: 10.1111/ger.12822 (PMC12606394; doi:10.1111/ger.12822)
Supplement: Supplementary file 1 — Table S1.The main domains of the topic guide. These domains were only used to semistructure the interviews. [file GER-42-529-s001.docx]

**Supplementary Materials:** the main domains of the topic guide. These domains were only used to semi-structure the interviews.

| Main domains | |
| --- | --- |
| 1. Oral Hygiene | The interviewees' perspectives on oral hygiene, oral disease prevention, and self-care, including disease-related issues. |
| 1. Dental Treatments | The interviewees' experiences regarding visiting dental practitioners, including dentists and oral hygienists. This includes not only the consultation itself, but also visiting the practice, and waiting in the waiting room, for example. |
| 1. Organisation | The interviewees’ experiences with dental care organizations, including collaboration with other care practitioners or, for example, financial aspects. |
| 1. Education and Research | This included the interviewees' experience regarding knowledge of dental and other care practitioners regarding oral health in PD patients. Furthermore, this topic included participants’ interest in future research projects on oral health in PD patients. |

Note | PD=Parkinson’s disease
